# Performance of a linear peristaltic infusion pump during red blood cells administration and the influence of infusion rates

**DOI:** 10.1186/2197-425X-3-S1-A548

**Published:** 2015-10-01

**Authors:** DM Kusahara, M Pedreira, AM Avelar, MA Peterlini

**Affiliations:** Pediatric Nursing, Federal University of São Paulo, São Paulo, Brazil

## Introduction

Even with the technological development of infusion pumps, pediatric intensive care nurses are resistant to administer red blood cells (RBC) by linear peristaltic infusion pumps due to the supposed risk of cells damage, despite the need of accuracy in low flow rates. Patient safety can be compromised both by RBC quality decrease due to the pumping mechanism, as well as, by possible flow rate problems during the administration.

## Objectives

To describe the level of hemolysis biomarkers and the hemolysis ratio of RBC administered by one linear peristaltic infusion pump. To verify the influence of three infusions rates programmed in a linear peristaltic infusion pump on the level of plasma hemoglobin and the hemolysis ratio.

## Methods

An experimental study was accomplished with 3 RBC bags (CPDA-1 preservative) from different donors with storage time ranging from 12 to 31 days. The linear peristaltic infusion pump was studied in triplicate. The equipment has a vertical peristaltic mechanism that compresses the administration tube halfway to closure and use a specific infusion set. The infusion pumps were set at 10 ml/h, 100 ml/h and 300 ml/h. The hemolysis ratio (%), plasma hemoglobin (mg/dl), potassium (mmol/L), lactate dehydrogenase (U/L), and haptoglobin (g/L) were analyzed. Samples for analysis were collected directly from the RBC (C1) bags external ports, after free flow of the blood component on the infusion disposable set (C2) and after submitted to the studied rate (E), exception made to haptoglobin that was analyzed in C1 and E. Data obtained were analyzed according to mean ± standard deviation, ANOVA and t student tests (p≤0.05).

## Results

A total of 54 analyses were performed. The comparisons between C1, C2 and E biomarkers values and hemolysis ratio demonstrated no significant variation: plasma hemoglobin of 0.92 ± 0.22, 0.90 ± 0.21 and 0.94 ± 0.20 (p = 0.966); potassium 30.0 ± 2.6, 30.5 ± 2.6, 30.8 ± 3.8 (p = 0.459); lactate dehydrogenase 705.2 ± 207.4, 739.9 ± 239.6 and 778.3 ± 318.7 (p = 0.475); hemolysis ratio 0.136 ± 0.038, 0.127 ± 0.035, 0.136 ± 0.049 (p = 0.615). The haptoglobin level was statistically similar between C1 and E (70.2 ± 38.1; 70.2 ± 38.0; p = 0.993). Comparisons of the studied infusion rates evidence significant variations (p≤0.001) in plasma hemoglobin levels among 10ml/h (0.106 ± 0.013) and 300 ml/h (0.072 ± 0.018), and comparing 100 ml/h (0.098 ± 0.010) and 300 ml/h (p = 0.002), without variations (p = 0.164) between 10ml/h and 100 ml/h. The infusion rate did not influenced significantly the hemolysis ratio (p > 0.05), that was 0.145 ± 0.030 at 10 ml/h, 0.131 ± 0.032 at 100 ml/h, and 0.122 ± 0.053 at 300 ml/h.

## Conclusions

The overall analyzes of the studied biomarkers and hemolysis ratio of RBC demonstrated no significant alterations related to the peristaltic infusion pumps, and the infusion rate of 300 ml/h caused less variation on plasma hemoglobin than 10 ml/h and 100 ml/h.

## Grant Acknowledgment

FAPESP-Sao Paulo Research Foundation n.12/25284-9

